# Pilot study to evaluate tools to collect pathologist annotations for validating machine learning algorithms

**DOI:** 10.1117/1.JMI.9.4.047501

**Published:** 2022-07-27

**Authors:** Katherine Elfer, Sarah Dudgeon, Victor Garcia, Kim Blenman, Evangelos Hytopoulos, Si Wen, Xiaoxian Li, Amy Ly, Bruce Werness, Manasi S. Sheth, Mohamed Amgad, Rajarsi Gupta, Joel Saltz, Matthew G. Hanna, Anna Ehinger, Dieter Peeters, Roberto Salgado, Brandon D. Gallas

**Affiliations:** a United States Food and Drug Administration, Center for Devices and Radiological Health, Office of Science and Engineering Laboratories, Division of Imaging Diagnostics & Software Reliability, Silver Spring, Maryland, United States; b National Institutes of Health, National Cancer Institute, Division of Cancer Prevention, Cancer Prevention Fellowship Program, Bethesda, Maryland, United States; c Yale University Computational Biology and Bioinformatics, New Haven, Connecticut, United States; d Yale New Haven Hospital, Center for Outcomes Research and Evaluation, New Haven, Connecticut, United States; e School of Medicine, Yale Cancer Center, Department of Internal Medicine, Section of Medical Oncology, New Haven, Connecticut, United States; f Yale University, School of Engineering and Applied Science, Department of Computer Science, New Haven, Connecticut, United States; g iRhythm Technologies Inc., San Francisco, California, United States; h Emory University School of Medicine, Department of Pathology and Laboratory Medicine, Atlanta, Georgia, United States; i Massachusetts General Hospital, Boston, Massachusetts, United States; j Inova Health System Department of Pathology, Falls Church, Virginia, United States; k Arrive Bio LLC, San Francisco, California, United States; l United States Food and Drug Administration (FDA), Center for Devices and Radiologic Health, Office of Product Evaluation and Quality, Office of Clinical Evidence and Analysis, Division of Biostatistics, White Oak, Maryland, United States; m Northwestern University Feinberg School of Medicine, Department of Pathology, Chicago, Illinois, United States; n SUNY Stony Brook Medicine, Department of Biomedical Informatics, Stony Brook, New York, United States; o SUNY Stony Brook Medicine, Department of Pathology, Stony Brook, New York, United States; p Memorial Sloan Kettering Cancer Center, New York, New York, United States; q Lund University, Laboratory Medicine, Region Skåne, Department of Genetics and Pathology, Lund, Sweden; r Sint-Maarten Hospital, Department of Pathology, Mechelen, Belgium; s University of Antwerp, Department of Biomedical Sciences, Antwerp, Belgium; t Peter Mac Callum Cancer Centre, Division of Research, Melbourne, Australia; u GZA-ZNA Hospitals, Department of Pathology, Antwerp, Belgium

**Keywords:** digital pathology, reader studies, reader variability, trothing, artificial intelligence, machine learning

## Abstract

**Purpose:**

Validation of artificial intelligence (AI) algorithms in digital pathology with a reference standard is necessary before widespread clinical use, but few examples focus on creating a reference standard based on pathologist annotations. This work assesses the results of a pilot study that collects density estimates of stromal tumor-infiltrating lymphocytes (sTILs) in breast cancer biopsy specimens. This work will inform the creation of a validation dataset for the evaluation of AI algorithms fit for a regulatory purpose.

**Approach:**

Collaborators and crowdsourced pathologists contributed glass slides, digital images, and annotations. Here, “annotations” refer to any marks, segmentations, measurements, or labels a pathologist adds to a report, image, region of interest (ROI), or biological feature. Pathologists estimated sTILs density in 640 ROIs from hematoxylin and eosin stained slides of 64 patients via two modalities: an optical light microscope and two digital image viewing platforms.

**Results:**

The pilot study generated 7373 sTILs density estimates from 29 pathologists. Analysis of annotations found the variability of density estimates per ROI increases with the mean; the root mean square differences were 4.46, 14.25, and 26.25 as the mean density ranged from 0% to 10%, 11% to 40%, and 41% to 100%, respectively. The pilot study informs three areas of improvement for future work: technical workflows, annotation platforms, and agreement analysis methods. Upgrades to the workflows and platforms will improve operability and increase annotation speed and consistency.

**Conclusions:**

Exploratory data analysis demonstrates the need to develop new statistical approaches for agreement. The pilot study dataset and analysis methods are publicly available to allow community feedback. The development and results of the validation dataset will be publicly available to serve as an instructive tool that can be replicated by developers and researchers.

## 1 Introduction

Recent advancements in the fields of digital pathology and artificial intelligence and machine learning (AI/ML) have the potential to transform the field of pathology by increasing the speed and accuracy of diagnosis.^1^^–^^5^ However, before implementing novel AI/ML algorithms for clinical use, their performance must be validated against a reference standard (ground truth) to assess their accuracy.^6^ In cases where an appropriate independent reference standard is not available, the reference standard may be established by human experts. Using human experts as a reference standard occurs in fields that rely on the interpretation of multi-factored visual information, like pathology.^5^^,^^7^

Using pathologist annotations as a reference standard is susceptible to interobserver variability.^8^^–^^10^ Here, as is often done in pathology, we use “annotations” to refer to any marks, segmentations, measurements, or labels a pathologist adds to a report, image, region of interest (ROI), or biological feature/target. Potential sources of variability in pathologist annotations include preanalytic and analytic variables. Preanalytic sources may include variability from tissue acquisition, tissue processing, tissue section thickness, tissue staining, slide digitization, and viewing digital displays.^11^^–^^13^ Analytic variability may result from differences in knowledge, residency training, subspecialty expertise, and experience as a practicing pathologist.^7^^,^^14^ This variability is compounded in validation studies when these differences are not standardized or captured in the data-collection process, which is further complicated by variable numbers of annotating pathologists between studies.^15^ Moreover, there is a lack of consensus agreement on the statistical methods for calculating interpathologist variability, which makes determining whether an AI/ML algorithm meets the acceptance criteria of performance difficult to ascertain. Therefore, there is a need to establish standardized workflows for efficiently collecting and using pathologist annotations and to construct the statistical methods needed to establish agreement between pathologists.

One example of where AI/ML algorithms may improve pathologist evaluation is in the estimation of the density of stromal tumor-infiltrating lymphocytes (sTILs) in triple-negative breast cancer (TNBC). sTILs density is estimated by pathologist assessment of hematoxylin and eosin (H&E) stained tumor-associated stromal tissue.^16^^–^^18^ In TNBC, the density of sTILs is a quantitative, prognostic, and predictive biomarker.^19^^–^^22^ High levels of sTILs density correlate with improved survival without and with therapy and predict positive response to therapy.^19^^,^^23^^–^^25^ Additional evidence indicates that the density of sTILs is a viable biomarker for other tumor types, such as nonsmall cell lung cancer, colorectal cancer, and prostate cancer.^17^^,^^26^^–^^29^ Yet, density evaluations of sTILs by pathologists are difficult and have notable variability, which makes developing a validation dataset for AI/ML algorithms for sTILs density estimation difficult.^26^^,^^30^^–^^32^ While flow cytometry, immunohistochemistry evaluation, and quantitative multiplex immunofluorescence also characterize sTILs in tissue, these approaches can be complex, time-consuming, expensive, and consume large quantities of tissue compared with estimating sTILs density in H&E slides during routine diagnostic examination.^29^^,^^33^^,^^34^ Therefore, a validation dataset using pathologist annotations on H&E slides will be accessible to the largest number of users. One method to develop such a validation dataset is to collect annotations from multiple pathologists on multiple cases to understand and improve the reference standard.

To maximize the efficiency of the annotation process, we proposed a standardized workflow and methodology to create a validation dataset of pathologist-annotated sTILs density estimates in H&E stained breast cancer biopsy slides.^35^ We launched a pilot study in February 2020 and collected pathologist annotations on an optical microscope platform and two digital whole slide image (WSI) web-viewer platforms through May 2021. The microscope platform (eeDAP: an evaluation environment for digital and analog pathology) is the reference technology.^36^ Pathologists are familiar with this technology, and it does not suffer from the limitations of slide digitization. In addition to the microscope platform, we used two digital image viewing and manipulation platforms, PathPresenter^37^^,^^38^ and caMicroscope.^39^ These platforms enable data collection worldwide regardless of physical proximity to the slides. An overview of this work can be found in an earlier publication.^35^

This manuscript presents initial results and analyses from a pilot study of the high throughput truthing (HTT) project. We discuss modifications to the original protocol and data-collection platforms made during and since the pilot study. These modifications were necessary as gaps were identified from pathologist feedback and data analysis. Such changes are the purpose of a pilot study and would not be permitted in a pivotal study. We also explore the validity of our quantitative assessment methods using the annotations collected on the PathPresenter digital platform, which will inform the development of statistical methods for interpathologist agreement using annotations. This assessment will inform the creation of a pivotal validation dataset (slides, images, annotations, and statistical methods) for the evaluation of AI/ML algorithms in digital pathology fit for a regulatory purpose that can be replicated by other researchers and developers.

## 2 Methods

### 2.1 Purpose of the Pilot Study

The purpose of our pilot study (or any pilot study) is to have an opportunity to evaluate the workflows and tools that will be used in the final pivotal study. Modifications to protocols and data-collection platforms can be made during and after the pilot study without spoiling the goals and hypotheses of a pivotal study. Here, we discuss issues, gaps, and inconsistencies identified from pathologist feedback and data analysis. We also discuss the specific changes made to our original protocol and data-collection tools to address these issues.

### 2.2 Clinical Use Case: sTILs Density Estimates

The clinical use case of this work is the annotation of sTILs in breast cancer H&E slides. We digitized 64 slides of invasive ductal carcinoma core biopsies prepared at a single site by recutting sections from formalin-fixed paraffin-embedded blocks. The slides were scanned on a Hamamatsu Nanozoomer-RS 2.0 (0.23  μm/pixel=40× magnification). Each slide represents a single case of breast cancer. A collaborating pathologist preselected ten 500  μm×500  μm regions of interest (ROIs) per slide for 640 total unique ROIs. The ROIs were selected to sample tumor and nontumoral tissue.^35^ For this pilot study, the number of ROIs was selected to provide both a representation of breast cancer tissue and sTILs variability and serve as the basis for sizing the pivotal study. The 64 cases in the pilot study were subdivided into eight batches of eight cases each with ten ROIs per case. Therefore, each batch consisted of 80 ROIs, and the full study had 640 ROIs. This subdivision into batches allowed for easier workload management and slide handling.

Pathologists annotated each ROI by providing comments on the sTILs content through a series of input tasks. The original description of the pilot study included two annotation tasks: label the ROI and, if the ROI is of a type that is evaluable for sTILs, estimate the density of sTILs (0% to 100%). The primary characteristic for an ROI to be evaluable is whether it contains tumor-associated stroma. With assistance from our collaborating pathologists, we created four options for labeling the ROI. Two of the label categories (“intratumoral stroma” and “invasive margin”) are considered evaluable for sTILs density estimation.^16^^,^^40^ If a pathologist labels an ROI with the other two labels (“tumor with no intervening stroma” or “other region”), the ROI is not evaluable for sTILs.

We added the task of estimating the percent of tumor-associated stroma after the launch of the pilot study. Percent tumor-associated stroma enables evaluation of an algorithm’s ability to identify tumor-associated stroma, the tissue where sTILs density is to be evaluated. This is a modification from the protocol previously described.^35^

The sTILs density is the percent of area of tumor-infiltrating lymphocytes within the area of tumor-associated stroma.^16^ It is calculated as

$$\mathrm{sTIL}s\;\mathrm{Density}=(\frac{\mathrm{Area}\;\mathrm{of}\;\mathrm{sTILs}}{\mathrm{Area}\;\mathrm{of}\;tu\mathrm{mor}\text{-}\mathrm{associated}\;\mathrm{stroma}})\times 100\%.$$

Following emerging evidence and recommendations from clinical experts, the sTILs density estimates can be grouped into three bins corresponding to anticipated patient management decisions and outcomes: “low” or 0% to 10%; “moderate” or 11% to 40%; and “high” or 41% to 100%.^18^^,^^30^^,^^31^ We provided pathologists with a visual reference created by Salgado et al.^16^ to help pathologists calibrate their estimates of the sTILs density. The reference sheet pairs representative ROIs of a range of sTILs densities in H&E images with a black-and-white image of that ROI with the sTILs digitally sectioned from the background content.

### 2.3 Recruitment of Readers and Description of Data Collection Technology

**Fig. 1:**
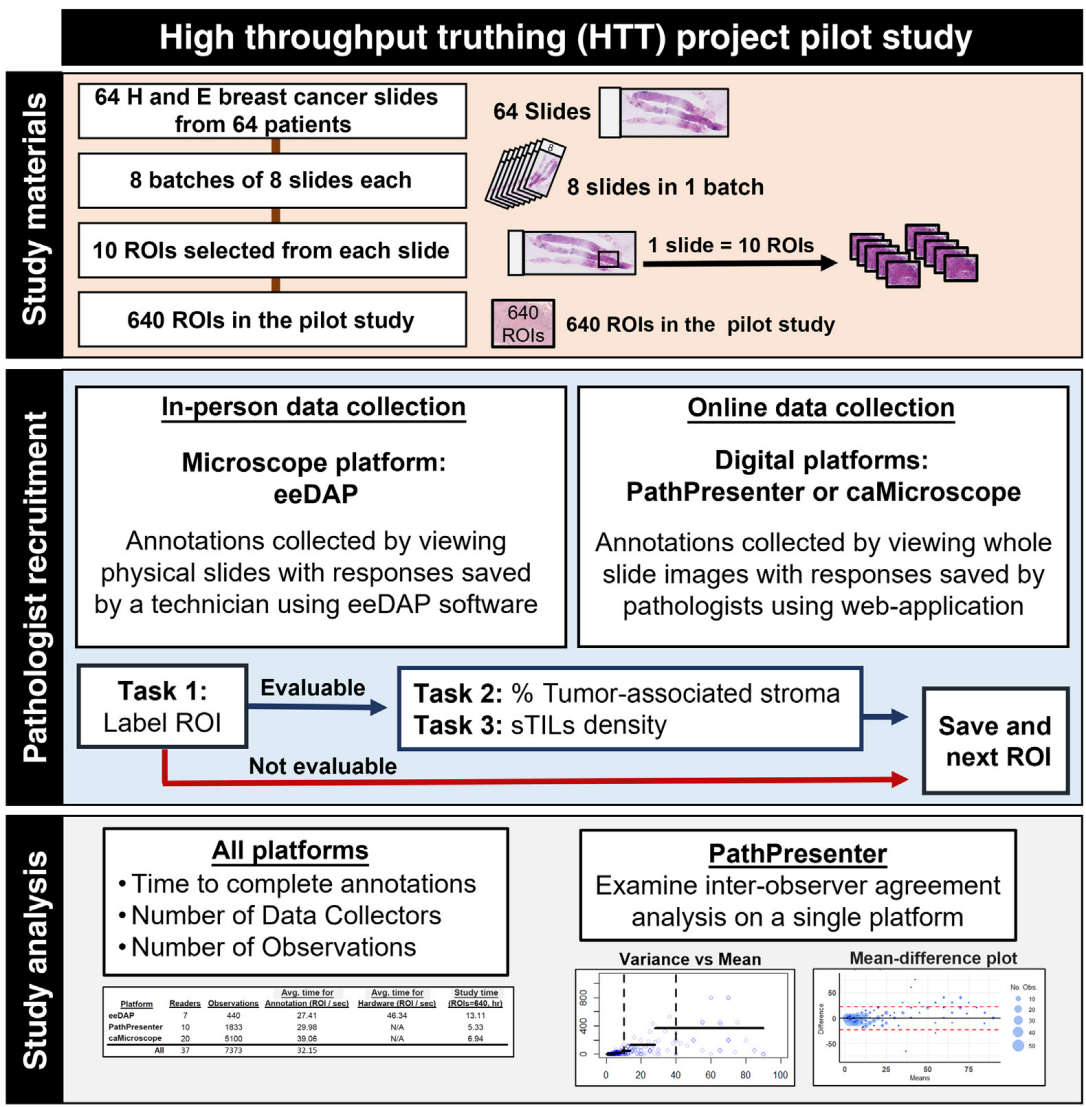
Workflow for the pilot study of the HTT Project. Study materials: the project includes a study of 640 ROIs from 64 breast cancer H&E slides. Pathologist recruitment: pathologists were recruited to either collect data in-person (eeDAP) and/or using a digital web-application (PathPresenter or caMicroscope). Study analysis: the results of the pilot study were then analyzed across all platforms and interobserver agreement analysis was evaluated using the results from the PathPresenter platform.

Following an IRB exempt determination protocol, we crowdsourced volunteer pathologists by recruiting at in-person conferences, online research forums, and emailing pathology communities for distribution to their networks. Crowdsourcing provides open access for data collection, enlisting the services of a large number of volunteers for the data collection process. These crowdsourced pathologists completed data-collection tasks via two modalities: an optical light microscope (eeDAP^36^) and two digital WSI viewing platforms (caMicroscope^39^ and PathPresenter^37^). Pathologists recruited in-person were directed to the eeDAP platform, and pathologists recruited online were directed to choose their preferred digital platform, PathPresenter, or caMicroscope. Pathologists were allowed to use multiple platforms during the pilot study. The workflow for the study is shown in Fig. 1.

Pathologists were recruited to contribute at an on-site data-collection event held in conjunction with the annual meeting of the United States and Canadian Academy of Pathology (USCAP) 2020. Further in-person data collection on eeDAP was paused due to the COVID-19 pandemic. The digital platforms replicated the eeDAP data-collection workflow and allowed continuous and world-wide data collection during the COVID-19 pandemic.

Using webinars, training videos, and scientific publications, we provided training on sTILs identification and density estimation. In total, ∼90  min of training was available for this study. We did not enforce completion of training prior to data collection. Pathologists were asked to annotate complete batches but were not required to annotate all 640 ROIs in the study. We also asked participants to complete batches of 80 ROIs in a random order, and late in the pilot study, we introduced a random-number-generator to assist pathologists with randomizing the sequence of batches. Use of the random-number-generator was not enforced.

### 2.4 Time to Collect Data Across All Three Platforms

We examined the time-associated burden of this study on pathologists. The time it takes to complete each ROI annotation is automatically recorded by the annotation software for all platforms. The time recorded by eeDAP does not include the time required for the microscope stage to move between ROIs. Additionally, the eeDAP system requires additional time to register each slide in a batch to the corresponding digital image. eeDAP consists of two levels of registration (low and high resolution) and a camera-eyepiece offset correction. The high-resolution registration and camera-eyepiece offset correction are optional but add additional time to the study. When these optional steps are performed, registration accuracy is ∼42  μm.^41^ We calculated the time for annotation on eeDAP by combining (1) the time it takes to set up and run through slides without deliberative annotation (an experiment completed by the study investigators, not the study pathologists) and (2) the average time it took study pathologists to annotate ROIs on eeDAP.

### 2.5 Quantitative Analysis of the PathPresenter Pilot Study Data

For this work, we analyzed the distribution of sTILs annotations from the PathPresenter digital platform collected from February 2020 to May 2021. Variability from platform differences were eliminated by analyzing only PathPresenter annotations. In this analysis, we calculated the sample mean and variance of the sTILs density estimates over the readers for each ROI, and we examined these variance estimates and corresponding coefficients of variation (CV) across the entire range and within the three density bins defined above (low, moderate, and high).

We also characterized the interpathologist agreement with the root-mean-squared difference, which is similar to the root-mean-squared error but does not require an independent reference standard.^42^ Specifically, the pathologist-to-pathologist expected mean-squared difference (MSD) is given as

$$\mathrm{MSD}=E[(X_{j^{\prime }kl}-X_{jkl}{)}^{2}],$$

where the X’s denote the sTILs density estimates from two different pathologists (j and $j^{\prime }$) for the same ROI (k and l denote the case and ROI, respectively). This metric is an average over pathologists, cases, and ROIs. In a full multireader and multicase study, we should account for the variability and correlations from readers and cases,^43^ as well as the correlation of ROIs that are nested within a case. In this work, we analyze two readers who each completed the entire study and ignore the correlation of ROIs.

### 2.6 Pilot Study Feedback from Expert Collaborators

After collecting and exploring the pilot study data, we convened an expert panel of seven practicing pathologists and one clinical scientist with expertise in microscopy and pathologist data collection.^44^ All expert panelists were pilot study collaborators with expertise in breast cancer pathology, training in sTILs assessment, and involvement in the design of our pilot study. One of these pathologists completed the pilot study. Two other expert pathologists fully annotated batch 1 (80 ROIs) of the pilot study. These annotations were completed at least 6 months before the expert panel was convened; crowdsourced pathologists were not invited. Through a series of eight recorded one-hour virtual sessions, the experts discussed each ROI regarding their sTILs assessment and experience with the data-collection process. The virtual sessions included a minimum of three expert panelists with study investigators guiding the discussion and note-taking. From these discussions, we identified limitations in our platforms and workflows. We also discussed potential resolutions to these limitations that will improve the pivotal study.^44^

## 3 Results

From the start of the pilot study in February 2020 through May 2021, 29 pathologists generated 7373 annotations across the three annotation platforms, with eight pathologists annotating on multiple platforms. Information about the readers in the study varies based on completion of the data-collection registration form. The registration form was provided to readers but not mandated. From the registration survey, 19 readers were board-certified pathologists with a range of 3 to 44 years since certification (median = 11 years and IQR = 17.5). Four readers reported current enrollment in a pathology residency program. Six readers did not provide information regarding their level of experience.

Annotations varied across platforms by number of ROIs scored and whether a pathologist completed a full study. By the conclusion of the pilot study, seven pathologists annotated all 640 ROIs on at least one platform. On PathPresenter, 50.2% (321/640) of the ROIs were annotated more than two times and two pathologists completed the full study (640 ROIs). Annotation within the study was uneven. The collection of the first 80 ROIs known as batch 1 was annotated 16 times, but all other batches were only annotated seven to nine times.

Table 1 shows the results of the data-collection efforts of the pilot study across all platforms. Information on total time to complete a study was estimated from available data. A study of the eeDAP platform examined the additional time needed to complete the study in-person due to slide registration and hardware operation. On average, the additional time needed for in-person data-collection was 30:54 min per four slides.

**Table 1:** Tabulated annotation counts and average annotation time of the pilot study by data collection platform. Included are the number of readers and individual observations per platform, the average time to annotate a single ROI per platform with standard deviation in brackets, additional time needed for eeDAP hardware operation, and the estimated total average time for a pathologist to complete a full study (n=640 ROIs). Values for batch time and study time are calculated by multiplying average time for annotation per ROI (ROI/sec) and average time for hardware accounting for the number of ROIs. As no full study set was completed on the eeDAP platform, these numbers are estimated from a small subset of readers (nReaders = 7 and nROI 80 to 240).

Platform	Readers	Observations	Avg. time for annotation [st. dev] (ROIs = 1, s)	Avg. time for hardware (ROIs = 1, s)	Batch time (ROIs = 80, h)	Study time (ROIs = 640, h)
**eeDAP**	7	440	27.41 [24.94]	46.34	1.64	13.11
**PathPresenter**	10	1833	29.98 [29.37]	N/A	0.67	5.33
**caMicroscope**	20	5100	39.06 [34.73]	N/A	0.87	6.94
**All**	37	7373	32.15 [35.52]			

We investigated the distribution of annotations across all platforms and summarized total ROIs stratified by tumor classification and sTILs density bin in Table 2. We tallied the instances for each of the four ROI labels and the mean sTILs density per ROI within each of the pre-selected density bins. The distribution was counted within individual platforms and summarized across all platforms. As shown in Table 2, over 75.8% (5592/7373) of all labels were listed as “intratumoral stroma.” Additionally, 60.9% (4492/7373) of all sTILs density estimates fell within the 0% to 10% and only 4.4% (322/7373) were estimates between 41% and 100%.

**Table 2:** Distribution of pathologist annotations (n=7373) in the pilot study by ROI label and the density bins. All ROIs are assigned an ROI label. If the label is considered evaluable, the pathologist then proceeds to assign the ROI a density estimate. Density estimates fell into one of three bins: low, moderate, or high.

	ROI label				Density Bins		
	Evaluable (n=5958)		Not evaluable		Low: 0% to 10%	Moderate: 11% to 40%	High: 41% to 100%
Platform	Intratumoral stroma	Invasive margin	Tumor with no intervening stroma	Other			
**eeDAP**	349	5	9	77	323	21	10
**PathPresenter**	1326	91	288	128	1127	211	79
**caMicroscope**	3917	270	210	703	3042	912	233
**All**	**5592**	**366**	**507**	**908**	**4492**	**1144**	**322**

**Fig. 2:**
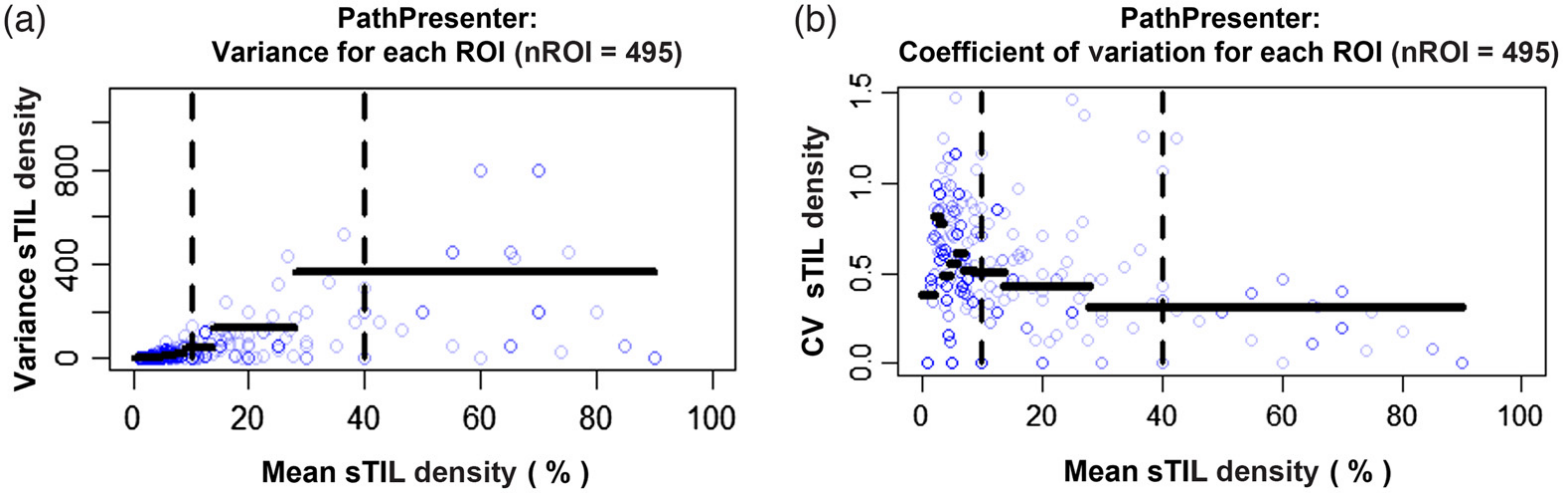
(a) Variance as a function of the mean calculated as averages over all readers on PathPresenter. Each blue circle represents one ROI (n=495) with at least two sTILs density estimates. The horizontal lines show the average variance in 10% bins of the data (49 ROIs). Vertical dashed lines split the data into low (0% to 10%), moderate (11% to 40%), and high (41% to 100%) sTILs density bins. (b) Coefficient of variation calculated as averages over all readers on PathPresenter.

We analyzed the sTILs density estimates for every ROI with at least two annotations on the PathPresenter platform (n=495). As four of the ten PathPresenter readers did not complete registration, we did not factor our analysis by experience level. In Fig. 2(a), we show the sample variance of the estimates as a function of the mean sTILs density estimates on the PathPresenter platform. From this figure, we observe that the pathologist variance depends on the ROI and increases with the mean. Figure 2(b) shows the CV of the sTILs density estimates as a function of the mean sTILs density estimates. We observe high CV at low sTILs density that decreases as the sTILs density increases. The CV plot also shows that the standard deviation of the data is not proportional to the mean.

We characterized interpathologist agreement by analyzing the annotations of the two pathologists who completed a full study on the PathPresenter platform. Table 3 shows the number of instances each pathologist gave a density estimate of an ROI or indicated an ROI was not evaluable. We analyzed the observations for each ROI as paired observations and averaged observations. For example, only instances where ROIs were marked as not evaluable by both pathologists were counted as a paired observation. Thus, 89 ROIs were labeled as not evaluable by both pathologists. In total, 448 ROIs were given density estimates by both pathologists (Table 3). This metric also helps us understand the number of ROIs assigned similar scores, but observations are lost when scores cross between density bins. Averaged observations are paired according to the mean density estimate of the ROI. Therefore, an ROI with density estimates across two bins (e.g., 9% and 11%) will be binned according to the mean of the estimates (10% = low-density bin). Finally, we calculated the root mean square difference (RMSD) within each density bin for the averaged observations.

**Table 3:** Individual and paired agreement analysis of the two pathologists who annotated all 640 ROIs in the study (anonymized ReaderIDs shown). The number of times each pathologist annotated an ROI as not evaluable or assigned a density estimate within the low, moderate, or high-density bin is shown in the first two rows. The third row shows the paired ROI observations for both pathologists. The fourth row shows the averaged observations for both pathologists. The last row is the calculated root mean square difference (RMSD) between the two pathologists across all densities and within the low, moderate, and high-density bins.

	Not evaluable	All densities: 0% to 100%	Low densities: 0% to 10%	Mod. densities: 11% to 40%	High densities: 41% to 100%
**ReaderID 4776**	92	548	426	94	28
**ReaderID 0455**	189	451	344	68	39
**Paired observations**	89	448	313	44	24
**Averaged observations**	N/A	448	319	93	36
**RMSD**		**10.62**	**4.62**	**14.25**	**26.25**

**Fig. 3:**
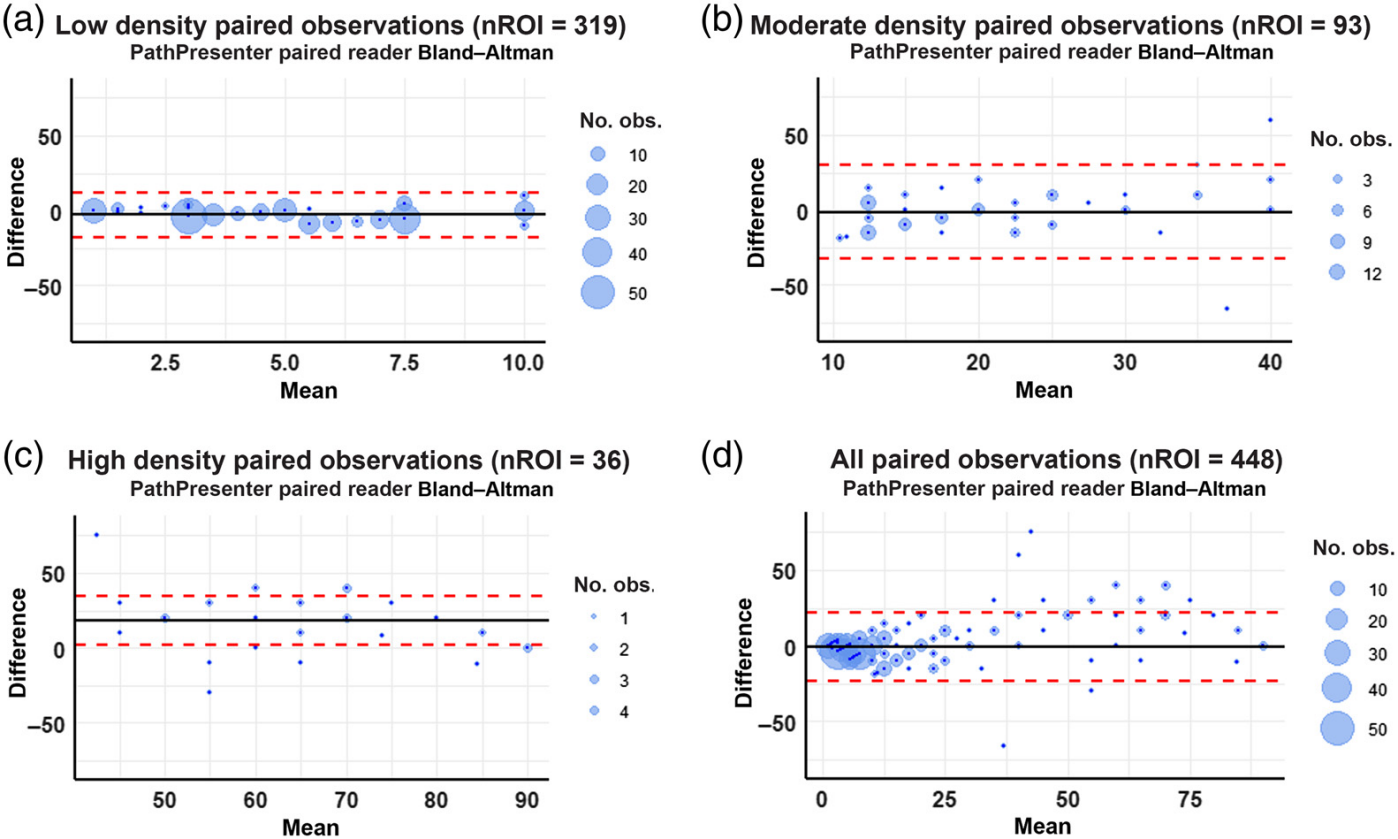
(a)–(d) Mean-difference (Bland–Altman) plots of the two pathologists with complete sets of annotations on the PathPresenter platform. Each blue circle represents a group of ROIs that share the same mean and different values. The size of the light blue circle is proportional to the number of ROIs; the largest circle corresponds to 50 ROIs. The horizontal black line shows the bias from 0 of each density bin. Red horizontal dashed lines indicate the upper and lower limits of agreement derived from the RMSD in Table 3.

Figure 3 shows Bland–Altman plots for each density bin to better visualize the difference between the two pathologists. In these plots, the differences between the paired observations are plotted against the mean of the estimates. The mean difference $\left(\overset{¯}{m}\right)$ for each bin is represented by the solid horizontal black line in each plot. The limits of agreement (horizontal red dashed lines) were constructed using twice the standard deviation of the differences in sTILs densities (RMSD2^−m¯2). As there were very few paired observations within the moderate and high-density bins, any further trend-identification analysis or confidence interval calculations were deemed inappropriate.

## 4 Discussion

The pilot study of the HTT project used three data collection platforms to generate 7373 annotations from 29 pathologists over a 15-month period. This manuscript summarizes the context, methods, and experience of the data collection with a focus on understanding processes to speed data collection and reduce pathologist variability. We focus on data collection and assessment time across platforms but only present quantitative the PathPresenter annotation results to understand pathologist variability on a single platform. Future work will look at the data from the other platforms, building on the lessons learned and presented here.

We estimated the time-burden on pathologists by a data-collection platform (Table 1). While the two digital platforms have similar estimated times (estimated average is 36 s/ROI), a pathologist using the eeDAP system can expect to spend double the amount of time on the study due to stage movements and registering the images to the slides. Thus, we are investigating upgrades to the eeDAP hardware and annotation workflow for the pivotal study. These time estimates are useful for planning future data collection activities and determining potential reimbursement rates for annotators.

We did not expect crowdsourced volunteer pathologists to annotate all 640 ROIs of the pilot study. The complete study takes more than five hours (Table 1), which is beyond reasonable expectations for most volunteers. Our plan for the pivotal study is to manage the workload of each pathologist by balancing the distribution of batches among the pathologists in the spirit of a split-plot study design.^45^^–^^47^ We did not effectively manage the workload of the pathologists in the pilot study as the data-collection tools did not yet have methods to create pathologist-specific work lists.

We found that batch 1 was annotated double the number of times as other batches. This finding suggests a need to either enforce the use of the random-number-generator or integrate another strategy to direct readers within the workflow. We analyzed the pilot study data as continuous data (0% to 100%) and within three predefined clinical bins (low 0% to 10%, moderate 11% to 40%, and high 41% to 100%) as prior studies of sTILs found associations between these density bins and clinical outcomes.^18^^,^^24^^,^^30^^,^^31^ We also observed 60.9% of the annotations were in the low sTILs density bin (Table 2). The two pathologists who annotated all 640 ROIs also identified over 70% of evaluable ROIs as low density (319/448), demonstrating that low-density ROIs persist throughout the study and are not confined to the most annotated batches (Table 4). These findings indicate that the ROIs selected for the pivotal study will need to avoid oversampling of the low-density bin within individual batches as well as within the study. Moreover, the entire pathologist workflow must be managed to ensure equitable distribution of annotations between batches.

Consistent with prior studies, there is high variability in pathologist sTILs density estimates (Fig. 2).^26^^,^^30^^–^^32^ Plotting variance as a function of the mean shows that the variance of the density estimates increases with the mean. We quantify this finding with the RMSD results in Table 3. The CV plot shows a complicated relationship with the mean with large relative variability for small sTILs densities (Fig. 2). Together, Figs. 2 and 3 indicate that the data are not independent and identically distributed or well-behaved as a function of the mean sTILs density. This precludes the use of many standard analysis methods for quantitative data. We have explored log and other transformations, but they did not stabilize the mean-variance relationship. As such, we will explore methods that treat the data as ordinal (ranks) and nominal (bins). Furthermore, the analyses in this work ignored the correlation between ROIs from the same slide. We will address this limitation in future work and properly account for those correlations.

This work identified several limitations in the technical workflows of the pilot study, including completion of the prestudy participant registration survey. The low completion rate for the pre-study registration translated to an inability to identify the experience level of 6 of the 29 pilot study readers. The original prestudy registration did not request information on a reader’s clinical specialty or familiarity with the clinical task, eliminating our ability to stratify expert readers from those new to the task or who had not completed training. These issues informed the design and integration of a mandatory registration survey that will prevent readers from starting the study until all fields of the registration survey are complete.

An additional component contributing to variability within the HTT project was the moderately structured and unenforced training provided to readers. As completion of the training modules was unmonitored and honor-based, we have no ability to determine the actual completion rates for study participants. We cannot evaluate the impact of existing training modules on pathologists’ annotations or extrapolate the potential reduction in variability resulting from enforcement and standardization of training. To resolve these issues, we are in the process of developing a standardized training module consisting of short videos describing the clinical task for each data collection platform, a training set of ROIs that will provide feedback on participant annotations, and a mandatory proficiency test. A passing grade on this proficiency test will be required to proceed with the study. Standardizing registration and training workflows will assist with evaluating pathologist performance in the pivotal study.

Finally, our data analysis results should be taken with caution. We made changes to the protocol and data-collection platforms during the pilot study. Another limitation of our analysis is the methods used to determine the average time needed for an annotation (Table 1). The eeDAP platform did not record the time related to microscope stage movements and both digital and microscope platforms can only determine the time spent on the annotation input screen (and will not reflect any interruptions). Nonetheless, we believe these measurements can help us plan the time it will take to conduct future pivotal studies, which is their true purpose here.

## 5 Conclusions

This work contributes to the field of digital pathology and AI/ML validation by establishing the methods and tools useful for the standardized collection of pathologist annotations. It also demonstrates that new analysis methods are needed as prior assumptions of the appropriate statistical methods for establishing interpathologist agreement were not supported, like treating the data as independent and identically distributed and finding variance differs as a function of the mean. This pilot study and the associated interpathologist agreement methods we are currently exploring will be used to size and create a validation dataset for AI/ML algorithms that yield sTILs density estimates in breast cancer. Our pilot dataset and analysis methods are available on a public HTT Github repository (https://github.com/didsr/htt) to allow open access to our methods and feedback from the digital pathology and statistics communities. The data and methods serve as an instructive tool for AI/ML algorithm developers and researchers and can be replicated for other tumor sites and quantitative biomarkers.

## Data Availability

The expert annotations on the select ROIs analyzed in this study will be publicly released before or in parallel with the publication of this work. The pilot study annotations are immediately available on this public repository: https://github.com/DIDSR/HTT.

## References

[1] Marble H. D. et al., “A regulatory science initiative to harmonize and standardize digital pathology and machine learning processes to speed up clinical innovation to patients,” J. Pathol. Inf. 11(1), 22 (2020). 10.4103/jpi.jpi_27_20 PMC7518200 33042601

[r2] Cucoranu I. C. et al., “Digital pathology: a systematic evaluation of the patent landscape,” J. Pathol. Inform. 5, 16 (2014). 10.4103/2153-3539.133112 25057430 PMC4060404

[r3] Cui M. Zhang D. Y., “Artificial intelligence and computational pathology,” Lab Invest. 101, 412–422 (2021). 10.1038/s41374-020-00514-0 33454724 PMC7811340

[r4] Bera K. et al., “Artificial intelligence in digital pathology - new tools for diagnosis and precision oncology,” Nat. Rev. Clin. Oncol. 16(11), 703–715 (2019). 10.1038/s41571-019-0252-y 31399699 PMC6880861

[5] Rashidi H. H. et al., “Artificial intelligence and machine learning in pathology: the present landscape of supervised methods,” Acad. Pathol. 6, 2374289519873088 (2019). 10.1177/2374289519873088 31523704 PMC6727099

[6] FDA/CDRH, Software as a Medical Device (SAMD): Clinical Evaluation, U.S. Food and Drug Administration (2017).

[7] Steiner D. F. Chen P.-H. C. Mermel C. H., “Closing the translation gap: AI applications in Digital Pathology,” Biochim. Biophys. Acta Rev. Cancer 1875, 188452 (2020). 10.1016/j.bbcan.2020.188452 33065195

[8] Janowczyk A. Madabhushi A., “Deep learning for digital pathology image analysis: a comprehensive tutorial with selected use cases,” J. Pathol. Inform. 7, 29 (2016). 10.4103/2153-3539.186902 27563488 PMC4977982

[r9] Dano H. et al., “Interobserver variability in upfront dichotomous histopathological assessment of ductal carcinoma in situ of the breast: the DCISion study,” Mod. Pathol. 33(3), 354–366 (2020). MODPEO 0893-3952 10.1038/s41379-019-0367-9 31534203 PMC7983551

[10] Tramm T. et al., “Standardized assessment of tumor-infiltrating lymphocytes in breast cancer: an evaluation of inter-observer agreement between pathologists,” Acta Oncol. 57(1), 90–94 (2018). 10.1080/0284186X.2017.1403040 29168428

[11] Engel K. B. Moore H. M., “Effects of preanalytical variables on the detection of proteins by immunohistochemistry in formalin-fixed, paraffin-embedded tissue,” Arch. Pathol. Lab. Med. 135(5), 537–543 (2011). APLMAS 0003-9985 10.5858/2010-0702-RAIR.1 21526952

[r12] Yildiz-Aktas I. Z. Dabbs D. J. Bhargava R., “The effect of cold ischemic time on the immunohistochemical evaluation of estrogen receptor, progesterone receptor, and HER2 expression in invasive breast carcinoma,” Mod. Pathol. 25(8), 1098–1105 (2012). 10.1038/modpathol.2012.59 22460807

[13] Khoury M. J. et al., “Health-centers for disease control and prevention multidisiplinary workshop,” Genet. Med. 11(8), 559–567 (2009). 10.1097/GIM.0b013e3181b13a6c 19617843 PMC2936269

[14] Allison K. H. et al., “Understanding diagnostic variability in breast pathology: lessons learned from an expert consensus review panel,” Histopathology 65(2), 240–251 (2014). HISTDD 1365-2559 10.1111/his.12387 24511905 PMC4506133

[15] Nagendran M. et al., “Artificial intelligence versus clinicians: systematic review of design, reporting standards, and claims of deep learning studies,” BMJ 368, m689 (2020). 10.1136/bmj.m689 32213531 PMC7190037

[16] Salgado R. et al., “The evaluation of tumor-infiltrating lymphocytes (TILs) in breast cancer: recommendations by an International TILs Working Group 2014,” Ann. Oncol. 26(2), 259–271 (2015). ANONE2 0923-7534 10.1093/annonc/mdu450 25214542 PMC6267863

[r17] Hendry S. et al., “Assessing tumor-infiltrating lymphocytes in solid tumors: A practical review for pathologists and proposal for a standardized method from the international immunooncology biomarkers working group: part 1: assessing the host immune response, TILs in invasive breast carcinoma and ductal carcinoma in situ, metastatic tumor deposits and areas for further research.,” Adv. Anat. Pathol. 24(5), 235–251 (2017). 10.1097/PAP.0000000000000162 28777142 PMC5564448

[18] Kos Z. et al., “Pitfalls in assessing stromal tumor infiltrating lymphocytes (sTILs) in breast cancer,” NPJ Breast Cancer 6(1), 17 (2020). 10.1038/s41523-020-0156-0 32411819 PMC7217863

[19] Mao Y. et al., “The prognostic value of tumor-infiltrating lymphocytes in breast cancer: a systematic review and meta-analysis,” Plos One 11(4), e0152500 (2016). POLNCL 1932-6203 10.1371/journal.pone.0152500 27073890 PMC4830515

[r20] Loi S. et al., “Tumor-infiltrating lymphocytes and prognosis: a pooled individual patient analysis of early-stage triple-negative breast cancers,” J. Clin. Oncol. 37(7), 559–569 (2019). 10.1200/JCO.18.01010 30650045 PMC7010425

[r21] Savas P. et al., “Clinical relevance of host immunity in breast cancer: from TILs to the clinic,” Nat. Rev. Clin. Oncol. 13(4), 228–241 (2016). 10.1038/nrclinonc.2015.215 26667975

[22] Denkert C. et al., “Tumour-infiltrating lymphocytes and prognosis in different subtypes of breast cancer: a pooled analysis of 3771 patients treated with neoadjuvant therapy,” Lancet Oncol. 19(1), 40–50 (2018). LOANBN 1470-2045 10.1016/S1470-2045(17)30904-X 29233559

[23] Park J. H. et al., “Prognostic value of tumor-infiltrating lymphocytes in patients with early-stage triple-negative breast cancers (TNBC) who did not receive adjuvant chemotherapy,” Ann. Oncol. Off. J. Eur. Soc. Med. Oncol. 30(12), 1941–1949 (2019). ANONE2 0923-7534 10.1093/annonc/mdz395 31566659

[r24] Luen S. J. et al., “Prognostic implications of residual disease tumor-infiltrating lymphocytes and residual cancer burden in triple-negative breast cancer patients after neoadjuvant chemotherapy,” Ann. Oncol. 30(2), 236–242 (2019). ANONE2 0923-7534 10.1093/annonc/mdy547 30590484

[25] Dieci M. V. et al., “Update on tumor-infiltrating lymphocytes (TILs) in breast cancer, including recommendations to assess TILs in residual disease after neoadjuvant therapy and in carcinoma in situ: a report of the International Immuno-Oncology Biomarker Working Group on Breast Cancer,” Semin. Cancer Biol. 52(Part 2), 16–25 (2017). SECBE7 1044-579X 10.1016/j.semcancer.2017.10.003 29024776

[26] Amgad M. et al., “Report on computational assessment of Tumor Infiltrating Lymphocytes from the International Immuno-Oncology Biomarker Working Group,” NPJ Breast Cancer 6, 16 (2020). 10.1038/s41523-020-0154-2 32411818 PMC7217824

[r27] Garutti M. et al., “Find the flame: predictive biomarkers for immunotherapy in melanoma,” Cancers 13(8), 1819 (2021). 10.3390/cancers13081819 33920288 PMC8070445

[r28] Uryvaev A. et al., “The role of tumor-infiltrating lymphocytes (TILs) as a predictive biomarker of response to anti-PD1 therapy in patients with metastatic non-small cell lung cancer or metastatic melanoma,” Med. Oncol. Northwood Lond. Engl. 35(3), 25 (2018). 10.1007/s12032-018-1080-0 29388007

[29] Gartrell R. D. et al., “Quantitative analysis of immune infiltrates in primary melanoma,” Cancer Immunol. Res. 6(4), 481 (2018). 10.1158/2326-6066.CIR-17-0360 29467127 PMC5882545

[30] Van Bockstal M. R. et al., “Interobserver variability in the assessment of stromal tumor-infiltrating lymphocytes (sTILs) in triple-negative invasive breast carcinoma influences the association with pathological complete response: the IVITA study,” Mod. Pathol. 34(12), 2130–2140 (2021). 10.1038/s41379-021-00865-z 34218258 PMC8595512

[r31] Swisher S. K. et al., “Interobserver agreement between pathologists assessing tumor-infiltrating lymphocytes (TILs) in breast cancer using methodology proposed by the International TILs Working Group,” Ann. Surg. Oncol. 23(7), 2242–2248 (2016). 10.1245/s10434-016-5173-8 26965699

[32] O’Loughlin M. et al., “Reproducibility and predictive value of scoring stromal tumour infiltrating lymphocytes in triple-negative breast cancer: a multi-institutional study,” Breast Cancer Res. Treat. 171(1), 1–9 (2018). BCTRD6 10.1007/s10549-018-4825-8 29774470

[33] Ju X. et al., “Predictive relevance of PD-L1 expression with pre-existing TILs in gastric cancer,” Oncotarget 8(59), 99372–99381 (2017). 10.18632/oncotarget.22079 29245908 PMC5725099

[34] Tan W. C. C. et al., “Overview of multiplex immunohistochemistry/immunofluorescence techniques in the era of cancer immunotherapy,” Cancer Commun. Lond. Engl. 40(4), 135–153 (2020). 10.1002/cac2.12023 PMC7170662 32301585

[35] Dudgeon S. et al., “A pathologist-annotated dataset for validating artificial intelligence: A project description and pilot study,” J. Pathol. Inf. 12(1), 45 (2021). 10.4103/jpi.jpi_83_20 PMC8609287 34881099

[36] Gallas B. D. et al., “Evaluation environment for digital and analog pathology (eeDAP): a platform for validation studies,” J Med Img 1(3), 037501 (2014). 10.1117/1.JMI.1.3.037501 PMC4478997 26158076

[37] AI PathPresenter, https://pathpresenter.net (accessed 16 Sept. 2020).

[38] Singh R., “Introducing PathPresenter, an innovative platform of pathology by pathologists for pathology practices,” Int. Acad. Pathol. Bull. 61(1), 4 (2021).

[39] Saltz J. et al., “A containerized software system for generation, management, and exploration of features from whole slide tissue images,” Cancer Res. 77(21), e79–e82 (2017). CNREA8 0008-5472 10.1158/0008-5472.CAN-17-0316 29092946 PMC5987533

[40] “Home,” Int. TILS Work. Group, https://www.tilsinbreastcancer.org/ (accessed 5 Nov. 2021).

[41] Gong Q. et al., “Registration accuracy between whole slide images and glass slides in eeDAP workflow,” Proc. SPIE 10581, 1058118 (2018). PSISDG 0277-786X 10.1117/12.2293189

[42] Obuchowski N. A., “Can electronic medical images replace hard-copy film? Defining and Testing the equivalence of dagnostic tests,” Stat. Med. 20(19), 2845–2863 (2001). SMEDDA 1097-0258 10.1002/sim.929 11568944

[43] Wen S. Gallas B. D., “Three-way mixed effect ANOVA to estimate MRMC limits of agreement,” Stat. Biopharm. Res. 1–10 (2022). 10.1080/19466315.2022.2063169

[44] Garcia V. et al., “Development of training materials for pathologists to provide machine learning validation data of tumor-infiltrating lymphocytes in breast cancer,” Cancers 14(10), 2467 (2022). 10.3390/cancers14102467 35626070 PMC9139395

[45] Obuchowski N. A. Gallas B. D. Hillis S. L., “Multi-reader ROC studies with split-plot designs: a comparison of statistical methods,” Acad. Radiol. 19(12), 1508–1517 (2012). 10.1016/j.acra.2012.09.012 23122570 PMC3522484

[r46] Gallas B. D. et al., “A collaborative project to produce regulatory-grade pathologist annotations to validate viewers and algorithms, in: abstracts,” J. Pathol. Inf. 10(1), 28 (2019). 10.4103/2153-3539.266902

[47] Chen W. Gong Q. Gallas B. D., “Paired split-plot designs in MRMC studies,” J. Med. Imaging 5(3), 031410 (2018). 10.1117/1.JMI.5.3.031410 PMC5956142 29795776

